# Supplementary material for: Tracking Charge Migration with Frequency-Matched Strobo-Spectroscopy
Source: J Phys Chem A. 2024 Jan 2;128(1):20–7. doi: 10.1021/acs.jpca.3c04234 (PMC10788909; doi:10.1021/acs.jpca.3c04234)
Supplement: Supplementary file 2 — jp3c04234_si_002.zip [file jp3c04234_si_002.zip › supplemental data/CM+FMSS_supplemental_data_documentation.pdf]

Supplemental data: Tracking Charge Migration with Frequency-Matched Strobo-Spectroscopy

Kyle A. Hamer, François Mauger, and Mette B. Gaarde

Department of Physics and Astronomy, Louisiana State University, Baton Rouge, LA 70803, USA

June 2, 2023

This documentation describes the time-dependent density-functional theory (TDDFT) data and Python scripts we use to make the figures published in "Tracking Charge Migration with Frequency-Matched Strobo-Spectroscopy." All the supplemental data were obtained with the Octopus package [Andrade 2012, Andrade 2015], version 11.4.

● Table of contents

● Table of contents 1
1 Octopus-package license 2
2 Supplementary data types 2
  2.1 Dipole signal files (tau_*) 2
  2.2 Charge migration files (CMdyn_*) 2
3 Supplementary data content 2
4 Python scripts 3
5 Acknowledgments 3
6 References 3

K.A. Hamer *et al.*, supplemental data documentation (2023).

1 Octopus-package license

This program is free software; you can redistribute it and/or modify it under the terms of the GNU General Public License as published by the Free Software Foundation; either version 2, or (at your option) any later version.

This program is distributed in the hope that it will be useful, but WITHOUT ANY WARRANTY; without even the implied warranty of MERCHANTABILITY or FITNESS FOR A PARTICULAR PURPOSE. See the GNU General Public License for more details.

2 Supplementary data types

Overall, we provide 2 types of supplementary-data files, with the naming conventions:

- `tau=*`: time-dependent dipole acceleration signal used to compute harmonic spectra.
- `CMdyn_*`: the corresponding laser field.

2.1 Dipole signal files (`tau_*`)

In each dipole signal file, the first column indicates the time (in atomic units), and columns 2-to-4 indicate the corresponding dipole acceleration in the x, y, and z directions, respectively. For further information on how the data is used to calculate the delay-dependent high-harmonic spectrum, see [Hamer 2023].

2.2 Charge migration files (`CMdyn_*`)

For a molecule aligned along the z axis, each file corresponds to the transverse-integrated density, at a given time t defined as

$$\rho_k(z,t) = \int_{box} dx\, dy\, |\psi_k(x,y,z,t)|^2,$$

where ψ_k is the kth Kohn-Sham orbital. In each file, the first two lines contain the time t and position z discretizations, and the remainder of the file contains the two-dimensional charge migration (CM) data $\rho_k(z,t)$. This file is most easily parsed by using the input_2d_paramdata() method found in utils.py -- see Section 4.

3 Supplementary data content

The supplementary data .zip file contains:

- *CM Dynamics*: the file `CMdyn_CMorbital.txt` contains the time-dependent density of the Kohn-Sham orbital in which an electron is removed to induce charge migration, used in Fig. 2(b) in the main text. The file `CMdyn_totalhole.txt` contains the density difference between the neutral and the time-dependent cation, or the hole density.
- *Dipole signal data:* the folder `dipoles/` contains three sub-directories:
    - `CM-orbital`: contains delay-dependent CM-orbital-resolved [Hamer 2021] dipole signals
    - `pi_x-system`: contains delay-dependent π_{xz}-orbitals-resolved dipole signals
    - `model`: contains delay-dependent model dipole signals [Hamer 2023]

4 Python scripts

The `Scripts/` directory contains the Python scripts we used to produce the figures in the paper. There are 3 scripts in total:

K.A. Hamer *et al.*, supplemental data documentation (2023).

- fig2.py
- figs3-5.py
- utils.py

The first 2 scripts produce the figures and subfigures of the paper, with self-explanatory names; the last script, utils.py, contains methods that are used throughout all of the scripts. If the scripts do not work on first execution, make sure that your integrated development environment (IDE) can locate utils.py. We have validated all the scripts on the Spyder IDE with Python 3.7.9.

5 Acknowledgments

This work was supported by the U.S. Department of Energy, Office of Science, Office of Basic Energy Sciences, under Award No. DE-SC0012462. Portions of this research were conducted with high performance computational resources provided by Louisiana State University (http://www.hpc.lsu.edu) and the Louisiana Optical Network Infrastructure (http://www.loni.org).

6 References

[Andrade 2012] X. Andrade, J. Alberdi-Rodriguez, D.A. Strubbe, M.J.T. Oliveira, F. Nogueira, A. Castro, J. Muguerza, A. Arruabarrena, S.G. Louie, A. Aspuru-Guzik, A. Rubio, and M.A.L. Marques, "Time-dependent density-functional theory in massively parallel computer architectures: the octopus project," J. Phys.: Condens. Matter 24, 233202 (2012).

[Andrade 2015] X. Andrade, D. Strubbe, U. De Giovannini, A.H. Larsen, M.J.T. Oliveira, J. Alberdi-Rodriguez, A. Varas, I. Theophilou, N. Helbig, M.J. Verstraete, L. Stella, F. Nogueira, A. Aspuru-Guzik, A. Castro, M.A.L. Marques, and A. Rubio, "Real-space grids and the Octopus code as tools for the development of new simulation approaches for electronic systems," Phys. Chem. Chem. Phys. 17, 31371 (2015).

[Hamer 2021] K.A. Hamer, D.R. Tuthill, T.D. Scarborough, L.F. DiMauro, K. Lopata, K.J. Schafer, M.B. Gaarde, and F. Mauger, "Orbital-resolved calculations of two-center interferences in linear triatomic molecules," Phys. Rev. A 104, 033114 (2021).

[Hamer 2023] K.A. Hamer, A. S. Folorunso, K. Lopata, K. J. Schafer, M. B. Gaarde, F. Mauger. "Tracking charge migration with frequency-matched strobo-spectroscopy," (in preparation, 2023).
